# A Scoring System to Predict Difficult Laparoscopic Cholecystectomy: A Five-Year Cross-Sectional Study

**DOI:** 10.1155/2022/3530568

**Published:** 2022-09-06

**Authors:** Agung Ary Wibowo, Oscar Tri Joko Putra, Zairin Noor Helmi, Hery Poerwosusanta, Tjahyo Kelono Utomo, Kenanga Marwan Sikumbang

**Affiliations:** ^1^Department of Surgery, Faculty of Medicine, Lambung Mangkurat University, Banjarmasin, Indonesia; ^2^Faculty of Medicine, Lambung Mangkurat University, Banjarmasin, Indonesia; ^3^Department of Orthopaedics and Traumatology, Faculty of Medicine, Lambung Mangkurat University, Banjarmasin, Indonesia; ^4^Department of Anesthesiology and Intensive Care, Faculty of Medicine, Lambung Mangkurat University, Banjarmasin, Indonesia

## Abstract

**Background:**

Laparoscopic cholecystectomy since long time already has become the preferred method because laparoscopic cholecystectomy has many advantages compared to standard open cholecystectomy. However, since it has associated with a higher risk of complication, preoperative prediction of risk factors is needed to assess the intraoperative difficulties. Various scoring systems have a role in predicting intraoperative difficulties; however, there is a need to find a consistent and reliable predictive system.

**Aim:**

To validate a preoperative scoring system that will predict difficult laparoscopic cholecystectomy. *Design of the Study.* Nonrandomized retrospective descriptive study. *Setting*. Department of General Surgery, Lambung Mangkurat Univeristy Ulin Referral Hospital, Banjarmasin, Kalimantan Selatan, Indonesia. *Methodology*. A preoperative score was given to all the patients (134 patients from January 2015–December 2020) based on history, clinical examination, and sonographic findings. Using ROC curve, the cutoff for easy—difficult was 3.5 and difficult—very difficult was 7.5. The scores were compared in each patient to conclude the practicality of the preoperative predictive score. SPSS version 25 was used to analyze the data.

**Results:**

History of hospitalization for acute cholecystitis (*p* ≤ 0.001), high BMI (*p*=0.002), abdominal scar (*p*=0.005), palpable gallbladder (*p* ≤ 0.001), thick gallbladder wall (*p* ≤ 0.001), and leucocyte (*p* ≤ 0.001) were considered as the significant factors that predict difficult laparoscopic cholecystectomy. Sensitivity and specificity for easy—difficult cutoff of the scoring method were 72.6% and 87.5%, respectively, with the area under the ROC curve being 0.849. Sensitivity and specificity for difficult—very difficult cutoff of the scoring method were 70.0% and 84.5%, respectively, with the area under the ROC curve being 0.779.

**Conclusion:**

The preoperative scoring system evaluated in the study is reliable and beneficial in predicting the difficulty of laparoscopic cholecystectomy. However, further randomized prospective multicentric studies with large sample sizes are required to validate the efficiency of the scoring system.

## 1. Introduction

Cholecystectomy is a surgical procedure to remove the gallbladder due to stone or inflammation, and its the most standard procedure performed in the biliary tract [1]. Laparoscopic cholecystectomy has become the preferred method and has been accepted as the gold standard for definitive management of symptomatic cholelithiasis or gallstones [1, 2]. This surgical procedure has many advantages over the standard open cholecystectomy, such as minimal trauma, decreased pain, shorter hospital stay, better cosmetic outcome, and faster recovery [3, 4]. However, some studies have shown that laparoscopic cholecystectomy has a higher frequency of complications than the standard open cholecystectomy. The complication includes injury of the common bile duct, bile leakage, gallbladder perforation, injury to the vascular and visceral structure during the application of Veress needle and a trocar, and other complications such as external biliary fistula, perihepatic collection, wound sepsis, hematoma, foreign body inclusions, adhesions, metastatic port-site deposits, and cholelithoptysis [1, 3]. In the early years of the laparoscopic cholecystectomy era, the conversion rate to open procedure was 2–15%. After years of learning and understanding the laparoscopic technique and increasing surgeons' experience, the conversion rate dropped to approximately 1–6%. This conversion was an attempt to avoid complications due to various difficulties encountered during the procedure [5]. The difficulty is considered in cases of dense adhesions at Calot's triangle, history of upper abdominal surgery, acutely inflamed and gangrenous gallbladder, empyema of the gallbladder, Mirizzi's syndrome, previous cholecystostomy, and cholecystogastric or cholecystoduodenal fistula [1].

Preoperative and intraoperative factors such as old age, body mass index (BMI), male gender, history of abdominal surgery, acute cholecystitis along with fever, leucocytosis, presence of gallbladder stones, and specific ultrasonographical findings such as distention of gallbladder and wall thickness ≥4 mm, impacted gallstones, and pericholecystic fluid collection are the risk factors that make laparoscopic cholecystectomy more complicated. Kama et al. reported a study that uses six parameters, such as old age, male gender, history of abdominal surgery, upper abdominal tenderness at the time of surgery, sonographically diagnosed thickened gallbladder wall, and the preoperative diagnosis of acute cholecystitis that was significantly associated with the risk of open cholecystectomy [1, 6].

To help surgeons decide on a surgical approach, counsel the patients, reduce the risk of complication, reduce the rate of conversion to open cholecystectomy, and reduce overall medical cost, a preoperative scoring system was made based on age, gender, history, clinical examination, laboratory, and sonographic findings and then it is compared with the score given based on intraoperative difficulties to predict the difficulty of laparoscopic cholecystectomy. Therefore, the present study was aimed to validate a scoring system to predict difficult laparoscopic cholecystectomy [7].

## 2. Patients and Method

We gathered data of five years (January 2015–December 2020) from a medical record, and the retrospective descriptive study was conducted at Ulin Referral Hospital. Each subject was observed for the following points: age, gender, history of hospitalization for acute cholecystitis, body mass index >27.5, previous abdominal surgery or abdominal scar, palpable gallbladder, thick gallbladder wall, and leukocytosis. The exclusion of this study was patients with jaundice.

### 2.1. Study and Procedure

**Figure 1:**
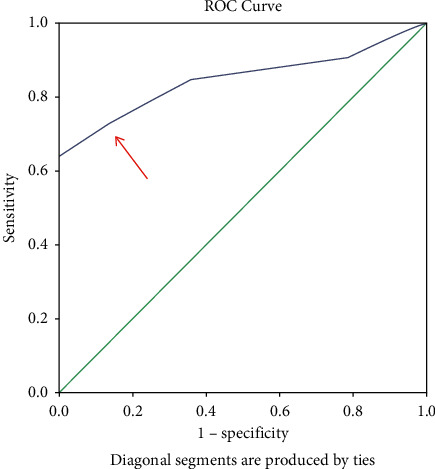
ROC curve 1 shows where to determine the cutoff for easy and difficult categories.

**Figure 2:**
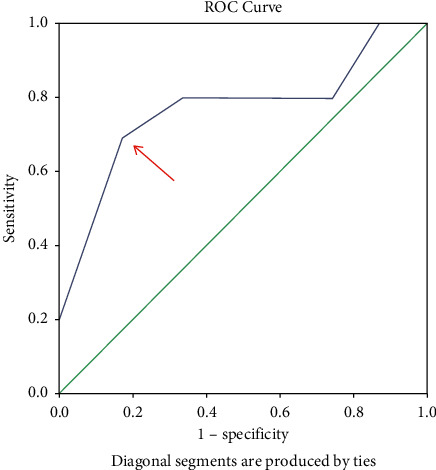
ROC curve 2 shows where to determine the cutoff for difficult and very difficult categories.

A preoperative score was given to all the patients based on history, clinical examination, and sonographic findings one day before the surgery (Table 1). ROC analysis was used to find the sensitivity and specificity of the scoring system to predict bailout procedure, then an optimal cutoff value was determined. From ROC curve 1, (see Figure 1) we get the optimum value for the cutoff value for the easy category, and the difficult variable preoperative score is 3.50 with a sensitivity number of 0.726 and a specificity number of 0.875, above or equal to the number is included in the difficult category, and below that number is included in the easy category. ROC curve 2 (see Figure 2) shows the optimum number for the cutoff value for the difficult and very difficult categories is obtained, the preoperative score variable is 7.50 with a sensitivity number of 0.700, and a specificity number of 0.845, above or equal to that number is included in the very difficult category, and below that number is in the difficult category (Table 2). Surgery was performed using carbon dioxide (CO_2_) pneumoperitoneum with 10 mmHg pressure and two 5 mm and 10 mm standard ports. Time was noted from first port-site insertion till final port closure. Intraoperative events such as duration of surgery were recorded, and surgery was labelled as easy/difficult/very difficult based on this duration of operation (Table 3). The intraoperative assessment was compared with a preoperative predictive score to determine the usefulness of the preoperative predictive score.

### 2.2. Statistical Analysis

Chi-square test tests were used to find the significant association between findings of the preoperative score and the intraoperative outcome. The area under the receiver operating characteristic (ROC) curve was used to find the diagnostic and predictive value of preoperative score for predicting the intraoperative outcome. *P* ≤ 0.05 was considered statistically significant.

## 3. Results

A total of 134 patients were involved in the study. Preoperative characteristics of the study patients are shown in Table 4. 80 (60%) of the patients were aged below 50 years, with a female preponderance 94 (70%). Of the 134 patients, 58 (43%) had a history of hospitalization for acute cholecystitis, 3 (2%) had BMI >27.5 kg/m^2^, 7 (5%) had an abdominal scar, 115 (86%) had palpable gall bladder, 60 (45%) had thick gallbladder wall, and 62 (46%) had leucocytosis (Table 4).

The Association of preoperative risk factors with the intraoperative outcome is shown in Table 5. History of hospitalization for acute cholecystitis (*p* ≤ 0.001), body mass index (*p*=0.002), abdominal scar (*p*=0.005), palpable gallbladder (*p* ≤ 0.001), thick gallbladder wall (*p* ≤ 0.001), and leucocyte (*p* ≤ 0.001) were considered as the significant factors that predict difficult laparoscopic cholecystectomy.

A comparison of preoperative score and outcome is shown in Table 6. Out of 40 easily predicted cases, 35 had easy laparoscopic cholecystectomies, five had difficult, and 0 had very difficult laparoscopic. Out of 84 predicted difficult cases, 23 had easy, 48 had difficult, and 13 had very difficult laparoscopic cholecystectomies. Out of 10 predicted difficult cases, two had easy, one had difficult, and 7 had very difficult laparoscopic cholecystectomies.

The relationship between the intraoperative outcome category and the preoperative score category was seen by chi-square correlation analysis, obtained a *p* value smaller than (0.000 < 0.050). It can be concluded that there is a significant relationship between the Intraoperative outcome category and the preoperative score category.

## 4. Discussion

Laparoscopic cholecystectomy has been considered the gold standard for the treatment of symptomatic gall stones [1, 2]. Intraoperative findings may not be similar in every case and could vary based on clinical presentation, and surgical difficulty might arise for the operating surgeon [8]. If the surgeons could predict the risk factors and safety of the procedure, surgeons could have benefit in deciding the surgical approach, counseling the patients, reducing the risk of complication, reducing the rate of conversion to open cholecystectomy, and reducing overall medical cost [7, 8]. In this study, laparoscopic cholecystectomy was performed in 134 patients, and various predictive risk factors for difficult laparoscopic cholecystectomy such as age, gender, history of hospitalization for acute cholecystitis, BMI, abdominal scar, palpable gallbladder, thick gallbladder wall in radiographic finding and leucocyte were analyzed. Each of these risk factors was reported to have a significant effect to predict difficult laparoscopic cholecystectomy by various studies [1, 3, 4, 7].

Increasing age has been considered a significant risk factor in predicting difficult laparoscopic cholecystectomy in various studies since the elderly population tends to have a higher likelihood of complicated biliary tract disease, which gets superimposed by various comorbidities [8–10]. However, this study found age did not affect the prediction of difficult laparoscopic cholecystectomy (*p*=0.996), which is correlated with other studies too [1, 8].

Previous studies have mentioned that gender is one of the significant risk factors, and the male population tends to have a high risk of conversion and surgical difficulty. In this study, gender was not a significant risk factor in predicting difficult laparoscopic cholecystectomy (*p*=0.157), which tolerates with studies which were conducted by Gupta et al. and Baral et al. that reported gender did not affect the prediction of difficulty in laparoscopic cholecystectomy. This could be due to less sample population of males in comparison to the female group [1, 8].

Patients with a history of hospitalization due to recurrence of acute cholecystitis had been shown to have high chances of difficult laparoscopic cholecystectomy due to repeated scarring, fibrosis, or dense adhesions at Calot's triangle and gallbladder fossa due to multiple colics, which has been clarified by this study too since we found this risk factor is significant in predicting the difficulty in laparoscopic cholecystectomy (*p* ≤ 0.001). Gupta et al. reported that these cases required more time for dissection of calot's triangle and dissection of gall bladder from the liver bed. Baral et al. stated in their study that the chances of difficulty that may lead to conversion are about six times higher than the patients who have not been previously admitted or treated conservatively for acute cholecystitis. A study by Stanisic et al. clarifies the absence of previous repeated attacks of cholecystitis and hospitalizations to determine the safeness of surgery [1, 8, 11].

Clinical findings such as BMI, abdominal scar, and palpable gallbladder were significant risk factors in predicting operative difficulties. From this study, BMI (*p*=0.002), abdominal scar (*p*=0.005), palpable gallbladder (*p* ≤ 0.001), thick gallbladder wall (*p* ≤ 0.001), and leucocytosis (*p* ≤ 0.001) were all significant risk factors in the prediction of a difficult surgery. Palpable gallbladder might be due to a thick-walled gallbladder, mucocele gallbladder, distended gallbladder, or due to adhesions between the gallbladder and the omentum. Similarly, studies conducted by Gupta et al. and Randhawa et al. found a significant association between the palpable gallbladder and intraoperative difficulty [9, 12].

Increased gallbladder thickness was another significant risk factor shown to predict difficult laparoscopic cholecystectomy because it could limit the extent of anatomical definition and could make dissection difficult at the gallbladder bed and Calot's triangle. In this study, a significant association was observed between gallbladder wall thickness and difficult laparoscopic cholecystectomy, similar to other studies in the literature. Another important ultrasonographic finding of the thick gallbladder wall was also a predictor of difficult laparoscopic cholecystectomy [13].

Using ROC curve, the cutoff for easy—difficult is 3.5, and difficult—very difficult is 7.5. This scoring method's sensitivity and specificity for easy—difficult cutoff were 72.6% and 87.5%, respectively, with the area under the ROC curve being 0.849. Sensitivity and specificity for difficult—very difficult cutoff of this scoring method were 70.0% and 84.5%, respectively, with the area under the ROC curve being 0.779. The correlation coefficient obtained is 0.573, which means that the relationship between the intraoperative outcome category and the preoperative score category is 0.50—0.75, which is a strong relationship category.

### 4.1. Limitation

This is a retrospective study. Therefore, some key statistics cannot be measured, and significant biases may affect the selection of controls. Researchers cannot control exposure or outcome assessment and instead must rely on others for accurate recordkeeping.

## 5. Conclusion

The preoperative scoring system evaluated in the study is reliable and beneficial in predicting the difficulty of laparoscopic cholecystectomy. However, further randomized, prospective, multicentric studies are required to validate the efficiency of the scoring system.

## Figures and Tables

**Table 1:** Preoperative scoring parameters used for grading the patient.

History	Level	Score	Max score
Age (years)	≤50 >50	0 1	1
Gender	Male Female	1 0	1
History of hospitalization for acute cholecystitis	Yes No	3 0	3

*Clinical parameters*	*Level*	*Score*	*Max score*
Body mass index (kg/m^2^)	<25 25–27.5 >27.5	0 2 3	3
Abdominal scar	No Infraumbilical Supraumbilical	0 1 2	2
Palpable gallbladder	Yes No	1 0	1

*Diagnostic test*	*Level*	*Score*	*Max score*
Sonographic: wall thickness	Thin ≤4 mm Thick ≥4 mm	0 2	2
Laboratory: leucocyte	<10.000/ul ≥10.000/ul	0 2	2

**Table 2:** ROC curve summary table.

State	Nilai cutoff	Sensitivity	1—specificity	AUC	Asymptotic Sig.
Easy—difficult	3.50	0.726	0.125	0.849	0.001
Difficult—very difficult	7.50	0.700	0.155	0.779	0.004

**Table 3:** Intraoperative assessment.

Parameters	Level
Operative time <60 min	Easy
Operative time 60–120 min	Difficult
Operative time >120 min	Very difficult

**Table 4:** Preoperative characteristics of the study patients.

Patient characteristics	*n* (%) *n* = 134
Age (years)	
≤50 >50	80 (60%) 54 (40%)

Gender	
Male Female	40 (30%) 94 (70%)

History of hospitalization for acute cholecystitis	
Yes No	58 (43%) 76 (57%)

Body mass index (kg/m^2^)	
<25 25–27.5 >27.5	103 (77%) 28 (21%) 3 (2%)

Abdominal scar	
No Infraumbilical Supraumbilical	127 (95%) 7 (5%) 0

Palpable gallbladder	
Yes No	115 (86%) 19 (14%)

Sonographic: wall thickness	
Thin ≤4 mm Thick ≥4 mm	74 (55%) 60 (45%)

Laboratory: leucocyte	
<10.000/ul ≥10.000/ul	72 (54%) 62 (46%)

**Table 5:** Relationship of risk factors with preoperative score.

Preoperative score factors	Level	Preoperative score			*r* (*p* value)
		Easy, *n* (%)	Difficult, *n* (%)	Very difficult, *n* (%)	
Age (years)	≤50	36 (26.9%)	32 (23.9%)	12 (9.0%)	0.007 (0.996)
	>50	24 (17.9%)	22 (16.4%)	8 (6.0%)	

Gender	Male	45 (33.6%)	33 (24.6%)	16 (11.9%)	0.164 (0.157)
	Female	15 (11.2%)	21 (15.7%)	4 (3.0%)	

History of hospital	Yes	56 (41.8%)	20 (14.9%)	0 (0.0%)	0.579 (0.000)^*∗*^
	No	4 (3.0%)	34 (25.4%)	20 (14.9%)	

Body mass index (kg/m^2^)	<25	52 (38.8%)	42 (31.3%)	9 (6.7%)	0.339 (0.002)^*∗*^
	25–27.5	8 (6.0%)	11 (8.2%)	9 (6.7%)	
	>27.5	0 (0.0%)	1 (0.7%)	2 (1.5%)	

Abdominal scar	Yes	59 (44.0%)	52 (38.8%)	16 (11.9%)	0.271 (0.005)^*∗*^
	No	1 (0.7%)	2 (1.5%)	4 (3.0%)	

Palpable gallbladder	Yes	59 (44.0%)	45 (33.6%)	11 (8.2%)	0.387 (0.000)^*∗*^
	No	1 (0.7%)	9 (6.7%)	9 (6.7%)	

Radiographic finding	Yes	52 (38.8%)	22 (16.4%)	0 (0.0%)	0.533 (0.000)^*∗*^
	No	8 (6.0%)	32 (23.9%)	20 (14.9%)	

Leucocyte	<10.000/ul	44 (32.8%)	24 (17.9%)	4 (3.0%)	0.363 (0.000)^*∗*^
	≥10.000/ul	16 (11.9%)	30 (22.4%)	16 (11.9%)	

^*∗*^Correlation significant.

**Table 6:** Summary table of relationship analysis on intraoperative outcome with preoperative score category with chi-square.

Preoperative score	Intraoperative outcome			Total, *n* (%)
	Easy (0–3.5)	Difficult (3.5–7.5)	Very difficult (7.5–15)	
Easy	35	5	0	40 (29.9%)
Difficult	23	48	13	84 (62.7%)
Very difficult	2	1	7	10 (7.5%)
Total, *n* (%)	60 (44.8%)	54 (40.3%)	20 (14.9%)	134 (100.0%)

*p* value = 0.000, *α* = 0.05, *p* value <*α*, *r* = 0.573.

## Data Availability

The data are available within the manuscript.
